# Characterization and Pathogenic Speculation of Xerostomia Associated with COVID-19: A Narrative Review

**DOI:** 10.3390/dj9110130

**Published:** 2021-11-10

**Authors:** Hironori Tsuchiya

**Affiliations:** School of Dentistry, Asahi University, Mizuho, Gifu 501-0296, Japan; tsuchi-hiroki16@dent.asahi-u.ac.jp; Tel.: +81-58-329-1263

**Keywords:** COVID-19, xerostomia, dry mouth, hyposalivation, prevalent, persistent, symptom characterization, pathogenesis

## Abstract

Patients with coronavirus disease 2019 (COVID-19) have become known to present with different oral symptoms. However, xerostomia remains poorly recognized compared with taste dysfunction. For better understanding of COVID-19 symptomatology, xerostomia associated withCOVID-19 was characterized and its possible pathogenesis was speculated by a narrative literature review. Scientific articles were retrieved by searching PubMed, LitCovid, ProQuest, Google Scholar, medRxiv and bioRxiv from 1 April 2020 with a cutoff date of 30 September 2021. Results of the literature search indicated that xerostomia is one of prevalent and persistent oral symptoms associated with COVID-19. In contrast to taste dysfunction, the prevalence and persistence of xerostomia do not necessarily depend on ethnicity, age, gender and disease severity of patients. COVID-19 xerostomia is pathogenically related to viral cellular entry-relevant protein expression, renin-angiotensin system disturbance, salivary gland inflammation, zinc deficiency, cranial neuropathy, intercurrent taste dysfunction, comorbidities and medications. Despite a close association with COVID-19, xerostomia, dry mouth and hyposalivation tend to be overlooked unlike ageusia, dysgeusia and hypogeusia. Although mouth dryness per se is not life-threating, it has an impact on the oral health-related quality of life. More attention should be paid to xerostomia in COVID-19 patients and survivors.

## 1. Introduction

Infection with severe acute respiratory syndrome coronavirus 2 (SARS-CoV-2) quickly spread worldwide together with the emergence of different variants with higher infectivity than the prototype virus, resulting in a global pandemic of coronavirus disease 2019 (COVID-19), causing 237,851,281 COVID-19 confirmed cases as of 11 October 2021 with over 4.8 million deaths according to the Johns Hopkins University and Medicine Coronavirus Resource Center [1]. Since the first report of SARS-CoV-2 infection in Wuhan, China in late 2019, nearly two years have passed, and accordingly, COVID-19 became known to exhibit a diverse spectrum of clinical manifestations, such as respiratory, neurological, cardiovascular, gastrointestinal and muscular ones [2,3]. Along with them, oral symptoms are becoming increasingly evident in COVID-19 patients and survivors [4,5]. Among oral symptoms, decreasing changes in saliva secretion result in dry mouth, xerostomia (subjective complaint of oral dryness) and hyposalivation (objective reduction of salivary flow rates, usually correlated with xerostomia).

In the literature of COVID-19 symptomatology, Freni et al. [6] first reported that patients infected with SARS-CoV-2 frequently complained of both taste dysfunction and xerostomia in the early phase of disease. In some cases, the prevalence of dry mouth was higher than or almost similar to that of impaired taste [7,8]. Compared with taste dysfunction, xerostomia remains poorly recognized despite the fact that not only dry mouth relates to COVID-19 [9] but also hyposalivation affects oral health and quality of life [10]. Hyposalivation has also been suggested to be a potential risk for SARS-CoV-2 infection [11].

However, there have been neither comprehensive studies of saliva secretion disorders in COVID-19 patients nor studies to elucidate the pathogenic mechanisms underlying such symptoms except for recent systematic reviews and meta-analyses of oral manifestations conducted by Aragoneses et al. [12] and Amorim dos Asantos et al. [13], which included xerostomia in COVID-19 patients. When the author previously reported oral symptoms associated with COVID-19 [4], only a small number of studies were found to deal with changes in saliva secretion of patients infected with SARS-CoV-2. Since then, the articles focusing on COVID-19 xerostomia have been gradually increasing. The aim of the present study is to characterize xerostomia, dry mouth and hyposalivation in COVID-19 patients and survivors and to speculate the possible pathogenesis for such symptoms by a narrative literature review in order to better understand COVID-19 oral stomatology.

## 2. Materials and Methods

Scientific articles were retrieved by searching PubMed, LitCovid, ProQuest and Google Scholar from 1 April 2020 with a cutoff date of 30 September 2021. Given the rapid world-wide spread of SARS-CoV-2 infection and the ever-progressing COVID-19 studies, the preprint data bases, medRxiv and bioRxiv, were also used to retrieve the latest information. The search was conducted by using the following terms or combinations thereof: “COVID-19”, “xerostomia”, “dry mouth”, “hyposalivation”, “oral symptom”, “saliva” and “salivary gland”. The exclusion criteria were papers that were not published in English and studies lacking demographic data. Cited papers in the retrieved articles were further searched for additional references. Collected articles were reviewed by title, abstract and text for relevance.

## 3. Results and Discussion

Results of the literature search indicated that xerostomia, dry mouth and hyposalivation are among frequent complaints of COVID-19 patients and survivors who were diagnosed by the reverse transcription-polymerase chain reaction (RT-PCR) test or according to the official guideline. Table 1 summarizes 12 relevant studies in order of the early publication date, including retrospective, prospective and case series studies. 

In addition to them, case reports of Tomo et al. [23], Eghbali Zarch and Hosseinzadeh [24], Díaz Rodríguez et al. [25] and da Mota Santana et al. [26] described that 37-, 56-, 78- and 85-year-old women complained of dry mouth or hyposalivation along with dysgeusia, burning tongue, lip sensation, angular chelitis and/or swallowing difficulty. Martelli Júnior et al. [27] analyzed the incidence in Brazil of Sjögren’s syndrome (inflammatory autoimmune disorder of the exocrine glands primarily resulting in oral and ocular dryness), and consequently revealed that Sjögren’s syndrome cases significantly increased following a COVID-19 pandemic. During the outbreak of SARS-CoV-2 infection, patients with the primary Sjögren’s syndrome (occurring independently of other health problems) reported a significant worsening in hyposalivation and salivary gland swelling [28].

The present study is a narrative literature review that may potentially be biased. Aragoneses et al. [12] assessed the risk of bias in prevalence of oral symptoms of COVID-19. In their assessment of prevalence studies, including COVID-19 xerostomia cited in the present study [7,14,16,17], the risk of bias was determined as low or moderate. The retrieved articles indicated that xerostomia appears in the early phase of COVID-19 to show prevalence ranging from 30% to 60% and that xerostomia can persist for at least 8 months after recovery from COVID-19. Amorim dos Santos et al. [13] conducted a meta-analysis of oral manifestations of 1017 COVID-19 cases and demonstrated that the overall prevalence of xerostomia was 43%. Eghbali Zarch and Hosseinzadeh [24] also analyzed 226 COVID-19 cases in 17 studies and suggested that dry mouth might be one of common oral symptoms, reported by 33.2% of COVID-19 patients.

In non-COVID-19 cases, risk factors of dry mouth are referred to as gender, age and medications taken [10]. The prevalence and persistence of xerostomia in COVID-19 cases may be similarly influenced by demographic data and symptom characteristics. Characterizing xerostomia while comparing with taste dysfunction will provide an insight into better understanding of oral symptoms in COVID-19 patients.

### 3.1. Xerostomia Characterization

#### 3.1.1. Ethnicity

In the early phase of COVID-19, 46.3% of 108 hospitalized Chinese patients developed dry mouth [8]. With respect to early cases in other countries, xerostomia and dry mouth were reported by 30–45.9% of 20–111 Italian patients with mostly mild to moderate COVID-19 [6,14,15], by 56.3–61.9% of 97–128 Israeli patients with mild COVID-19 [7,20] and by 60% of 10 hospitalized Iranian COVID-19 patients [19]. An ethnic difference of xerostomia is unlikely to be significant unlike taste dysfunction, which is more prevalent in European cohorts than in Asian cohorts [4,29].

In a cross-sectional study of Field et al. [30], the prevalence of xerostomia in outpatients attending a dental practice was lower in an English population than in North American and Swedish populations. While xerostomia is a common consequence of the radiotherapy in head and neck cancer, xerostomia occurring within 6 months after radiotherapy showed a racial difference in prevalence [31]. When evaluating clinical characteristics of patients with Sjögren’s syndrome, patients with a chief complaint of xerostomia were more likely to be white and Asian patients were less likely to complain of xerostomia compared with whites [32].

Unlike these non-COVID-19 cases, ethnicity does not necessarily determine the prevalence of xerostomia in the early phase of COVID-19. However, the number of comparative studies between different countries is too small to determine the ethnicity-independence of COVID-19 xerostomia.

#### 3.1.2. Age

In the early phase of COVID-19 or during hospitalization, prevalence of xerostomia or dry mouth was 32.0–61.9% for Italian, Israeli, Iranian and Egyptian patients younger than 50 years old [6,7,19,20,21] and 30.0–46.3% for Chinese and Italian patients older than 50 years old [8,14,15]. When following up after 4 weeks to 8 months from hospital discharge or symptom onset, xerostomia persisted in 14.4–40.2% of Turkish, Israeli and Colombian COVID-19 survivors younger than 50 years old [18,20,22] and in 24.6% of Italian COVID-19 survivors older than 50 years old [16]. Xerostomia was also reported by 47.6% of Egyptian patients aged mean 36.2 years who recovered from mild to moderate COVID-19 [17].

In non-COVID-19 cases, Niklander et al. [10] assessed xerostomia of 566 patients attending the Universidad Andrés Bello Dental School Clinic (Chile). While xerostomia was reported by 10.8% of patients, the highest prevalence was observed in patients aged 68 to 77 years (33.3%), followed by patients aged 78 to 83 years (22.27%). Field et al. [30] also comparatively determined the prevalence of xerostomia in 1103 adult patients (38.7% aged 60 years or older) attending for routine dental care. Their logistic regression analysis indicated that age was a significant risk factor for xerostomia.

Although the occurrence and prevalence of taste dysfunction vary by the age of COVID-19 patients [4], the age-dependence of COVID-19 xerostomia remains inconclusive because xerostomia and hyposalivation in non-COVID-19 populations are increasingly frequent with age, especially over 60 years. As described by Hopcraft and Tan [33], aging per se has no significant impact on salivary flow rates, but the prevalence of xerostomia increases in the middle-aged and elderly populations.

#### 3.1.3. Gender

Biadsee et al. [7] assessed sex-related symptoms of Israeli non-hospitalized patients in the early phase of COVID-19. Dry mouth was reported by 56.3% of 128 patients, in which 34.4% were females (prevalence of 62.9% in 70 females) and 21.9% were males (prevalence of 48.3% in 58 males), suggesting that xerostomia is more prevalent in females than males. Gherlone et al. [16] followed up 122 Italian hospitalized patients with moderate to severe COVID-19 after a mean 104 days from hospital discharge. In contrast to the early symptom, dry mouth persisted in males more frequently than in females. The overall prevalence was 19.7% in males (80% in 30 male COVID-19 survivors) and 4.9% in females (20% in 30 female COVID-19 survivors).

In the early phase of COVID-19, Chen et al. [8] collected subjectively-reported questionnaires from 108 Chinese hospitalized COVID-19 patients. Their data analysis indicated that prevalence of dry mouth was 46.3% overall, 46.4% in females and 46.2% in males, showing no statistically significant difference between female and male patients. AbuBakr et al. [17] and Omezli and Torul [18] assessed oral symptoms of 573 Egyptian patients who recovered from mild to moderate COVID-19 and 107 Turkish patients with mild to moderate COVID-19 who were discharged from hospital, respectively. Although xerostomia persisted in 40.2–47.6% of these COVID-19 survivors, the statistical analysis showed no significant differences in prevalence between females and males (*p* = 0.5 in the Egyptian cohort [17] and *p* = 0.928 in the Turkish cohort [18]). Biadsee et al. [20] followed up with 97 Israeli COVID-19 patients after 8 months from the RT-PCR negative results and demonstrated that 8.2% of female COVID-19 survivors and 6.2% of male COVID-19 survivors complained of xerostomia (14.8% in 54 females and 14.0% in 43 males, *p* = 0.905). These results suggest that gender difference does not necessarily influence the prevalence and persistence of COVID-19 xerostomia.

Gender has been referred to as an independent risk factor to cause xerostomia in non-COVID-19 cases. Niklander et al. [10] assessed oral symptoms of 566 patients attending dental practice and showed that xerostomia was reported by 61 patients (corresponding to 10.8%) consisting of 50 females (83.3%) and 11 males (16.7%). The prevalence of xerostomia was 13% in female patients but 6.1% in male patients. Field et al. [30] also revealed that females developed xerostomia more frequently than males in 1103 English adult patients attending for routine dental care.

Among oral symptoms associated with COVID-19, taste dysfunction is likely to depend on gender; its prevalence has been shown to be higher in female patients than male patients [3]. However, in contrast to non-COVID-19 cases, the prevalence and persistence of COVID-19 xerostomia in females and males varied from study to study. A case series study of Biadsee et al. [7] indicated no significant differences in prevalence of xerostomia between female and male COVID-19 patients.

#### 3.1.4. Disease Severity

Taste dysfunction is associated with relatively mild severity of COVID-19. In the early phase of COVID-19, patients with asymptomatic, mild, moderate, severe and critical disease have been shown to complain of ageusia in decreasing order of prevalence [4]. At a 6-month follow-up after COVID-19 onset of hospitalized and non-hospitalized patients, dysgeusia/anosmia was shown to persist in 13.9% of patients with mild disease, in 4.3% with moderate, in 4.2% with severe and in 0% with critical disease [34]. The severity of COVID-19 at baseline is likely to determine the prevalence and persistence of taste dysfunction.

When 107 Turkish COVID-19 patients whose treatment completed was completed for at least 2 weeks were evaluated for xerostomia persistence [18], no significant differences were observed in saliva flow between patients having treatment at home and hospital (*p* = 0.187). Anaya et al. [22] followed up with 100 Colombian COVID-19 patients with different disease severity and assessed their symptoms after 7 months from symptom onset. Xerostomia persisted in 25.7% of 35 ambulatory patients (mild COVID-19), in 19.5% of patients with severe COVID-19 and in 37.5% of patients with critical COVID-19 (*p* = 0.28). Their results suggest that xerostomia in COVID-19 survivors may not necessarily be influenced by the severity of disease at baseline, unlike with COVID-19 taste dysfunction.

#### 3.1.5. Association with Taste Dysfunction

Xerostomia in COVID-19 patients and survivors has been shown to be accompanied by taste impairments, oral mucosal ulcerations, swallowing difficulty, burning sensation and halitosis. Among such oral symptoms, xerostomia/dry mouth and taste dysfunction in the early phase of COVID-19 was reported by 30.0–45.9% and 25.0–70.0% of Italian patients [6,14,15], by 56.3–61.9% and 52.3–67.0% of Israeli patients [7,20], by 46.3% and 47.2% of Chinese patients [8] and by 39.7% and 34.5% of Egyptian patients [21], respectively. Xerostomia/dry mouth and taste dysfunction persisted in 40.2% and 56.1% of Turkish survivors after 2–4 weeks from hospital discharge [18], in 24.6% and 42% of Italian survivors at a mean 104-day follow-up after hospital discharge [16], in 26.0% and 53.0% of Colombian survivors at a 7.3-month follow-up after symptom onset [22] and in 14.4% and 25.8% of Israeli survivors at 6–8 month follow-ups after the RT-PCR test negativity [20], respectively. COVID-19 xerostomia is closely associated with taste dysfunction.

#### 3.1.6. Comorbidities

Seven of the 12 retrieved studies referred to comorbidities of COVID-19 patients and survivors. The most common comorbidities were diabetes mellitus and hypertension that were reported by 2.3–26.7% and 6.3–40% of the test subjects, respectively [6,7,14,15,16,22]. In addition, chronic pulmonary disease, thyroid disease and chronic kidney disease were found in 1.0–20.0%, 3.1–12.0% and 3.0–6.7% of COVID-19 patients, respectively.

### 3.2. Pathogenic Speculation

Xerostomia has been commonly considered to occur secondary to nasal congestion and rhinorrhea due to mouth breathing. However, Biadsee et al. [7] revealed that nasal congestion and rhinorrhea did not correlate to COVID-19 xerostomia. Xerostomia in COVID-19 patients and survivors may be due to multiple causes because different pathogenic mechanisms are presumed as they include viral cellular entry-relevant protein expression in salivary glands, renin-angiotensin system disturbance, salivary gland inflammation, zinc deficiency, cranial neuropathy, intercurrent taste dysfunction, comorbidities and medications. Okada et al. [35] recently published an excellent review on the pathogenesis of taste impairment and salivary dysfunction in COVID-19 patients.

#### 3.2.1. Viral Cellular Entry-Relevant Proteins

In SARS-CoV-2 infection, the viruses primarily bind to angiotensin-converting enzyme 2 (ACE2) receptors on host cells, followed by fusion of the viral lipid envelopes with cellular membranes of the host cells. The spike protein of SARS-CoV-2 consists of S1 and S2 subunits, in which the S1 subunit contains an ACE2 recognition motif in the receptor-binding domain and the S2 subunit is membrane-anchored and harbors the fusion machinery [36]. The viral S1 subunit initially binds to an ACE2 receptor on host cells. Subsequently, host cell protein convertase Furin cleaves the spike protein at the S1/S2 site (between S1 and S2 subunit), thereafter dissociating the S1 subunit from the spike protein. Host cell transmembrane serine protease 2 (TMPRSS2) cleaves at the S2′ site of the S2 subunit, enabling the resultant viral structure to fuse with host cell membranes [37]. Therefore, it is critical for SARS-CoV-2 to affect saliva secretion whether ACE2, TMPRSS2 and Furin are present in salivary glands.

Song et al. [38] analyzed the expression of ACE2 and TMPRSS2 in salivary glands in a healthy human population using the Genotype-Tissue Expression dataset. They suggested the possibility that ACE2 and TMPRSS2 may be expressed in salivary glands without significant differences in gender and age. Sakaguchi et al. [39] first demonstrated that ACE2, TMPRSS2 and Furin were expressed in human submandibular glands by performing a series of immunohistochemical experiments on the existence of SARS-CoV-2 cellular entry molecules in the oral cavity. They first demonstrated that ACE2, TMPRSS2 and Furin were expressed in human submandibular glands. Yoshimura et al. [40] immunohistochemically examined biopsied human salivary glands from non-COVID-19 patients with diseases of the oral and maxillofacial region. They revealed that ACE2-positive cells were present in major salivary glands (parotid and sublingual glands) and minor salivary glands (buccal glands). With respect to COVID-19 patients, Matuck et al. [41] conducted ultrasound-guided postmortem biopsies in 24 COVID-19 fatal cases to study salivary gland samples by quantitative reverse transcription-polymerase chain reaction (qRT-PCR), immunochemical assay and electron microscopic analysis. The qRT-PCR for SARS-CoV-2 was positive in 30 salivary glands from 18 patients and spherical 70–100 nm viral particles (consistent in size and shape with the *Coronaviridae* family) were detected in submandibular and parotid glands. Their immunohistochemical results indicated that ACE2 and TMPRSS2 were expressed in both parotid and submandibular glands. Soares et al. [42] also reported the high expression of ACE2 in minor salivary glands of COVID-19 patients.

Salivary glands structurally consist of acinar (serous and mucous) cells organizing into acini, ductal cells organizing into intercalated excretory ducts and myoepithelial cells surrounding acini and intercalated ducts to provide them with contractile support. SARS-CoV-2 cellular entry-relevant proteins have been revealed to be distributed in different subpopulations of cells within salivary glands. Huang et al. [43] examined COVID-19 autopsy salivary glands and showed that ACE2 was expressed in salivary gland ducts and serous and mucous acini and that SARS-CoV-2 mRNA was consistently detected in ACE2-expressing ducts and acini. They also observed that the virally infected acini and ducts harbored replicating SARS-CoV-2. By exploring human genomic and protein databases, Zupin et al. [44] indicated that Furin was expressed in submandibular salivary glands with its gene expression distributed in different cells. Zhu et al. [45] recently reported the salivary gland expression, cellular location and viral binding of ACE2 and TMPRSS2 after a series of experiments using major salivary glands from non-COVID-19 patients afflicted with benign disorders. They revealed that ACE2 and TMPRSS2 were expressed in parotid, submandibular and sublingual glands in decreasing order of expression level. Both SARS-CoV-2 cellular entry-relevant proteins were located in the cytoplasm and cytomembrane of serous acinar cells and duct epithelial cells of parotid and submandibular glands and in the cytoplasm and cytomembrane of serous acinar cells in the mixed acini of sublingual glands. Furthermore, they reached remarkable findings by confirming that SARS-CoV-2 spike proteins were able to bind to human parotid, submandibular and sublingual glands. These studies suggest that SARS-CoV-2 could invade major and minor salivary glands through the interactions with ACE2, TMPRSS2 and Furin expressed in both salivary glands. After the cellular entry via the receptor- and protease-mediated pathway, cytopathic SARS-CoV-2 possibly damages salivary glands and affects their secretory functions to cause xerostomia in the early phase of COVID-19. In humans, more than 90% of saliva is secreted by three major salivary glands, in which submandibular and sublingual glands are responsible for 68% of saliva and parotid glands for 28% at resting, and submandibular and sublingual glands are responsible for 46% of saliva and parotid glands for 53% during stimulation, whereas minor salivary glands are responsible for the remainder [46]. The pathogenic contribution of major salivary glands to COVID-19 xerostomia is considered more significant compared with minor salivary glands.

Turnover of saliva-producing acinar cells is estimated to range from 50 to 125 days and acinar cells are replaced within 6 months [47]. Once salivary glands are damaged by SARS-CoV-2, they need several months to recover the secretory functions, which could mechanistically underlie persistent xerostomia. In addition, long-term in vivo presence of SARS-CoV-2 may be another cause for persistent xerostomia in COVID-19 survivors. SARS-CoV-2 has the possibility to remain for a certain period in tissues of patients recovered from COVID-19. While viable viruses are successfully isolated in the first 8 days after symptom onset [48], prolonged SARS-CoV-2 RNA shedding after symptomatic relief is not rare as the longest duration of viral RNA detection in the respiratory tract was estimated to be 83 days [49]. SARS-CoV-2 potentially continues to replicate and spread in cells expressing ACE2 receptors [50].

#### 3.2.2. Renin–Angiotensin System Disturbance

The renin–angiotensin system (RAS) plays an important role in fluid and electrolyte homeostasis as well as in regulation of blood pressure. Central RAS components constitute the two main axes: (1) angiotensin-converting enzyme (ACE or ACE1) converts angiotensin I to angiotensin II that binds to angiotensin II type 1 receptor (AT1R) to stimulate angiogenesis and cell proliferation, and to angiotensin II type 2 receptor (AT2R); and (2) ACE2, a homologue of ACE, degrades angiotensin II to angiotensin (1–7) that acts on mitochondrial assembly receptor (MASR) to antagonize the angiotensin II/AT1R stimulation. The ACE2, angiotensin (1–7) and MASR axis counterbalances the ACE, angiotensin II and AT1R axis. SARS-CoV-2 can disturb the ACE/ACE2 balance and activate the RAS [51]. Cano et al. [52] immunohistochemically characterized the local RAS in major salivary glands of rats. Among RAS components, ACE, ACE2, AT1R, AT2R and MASR were found to be present in acinar, duct and myoepithelial cells and blood vessels of parotid, submandibular and sublingual glands.

ACE2 plays a dual role of in COVID-19, that is, ACE2 initially acts as a receptor for SARS-CoV-2 to enter host cells, and then, ACE2 is downregulated in the context of SARS-CoV-2 infection, resulting in an increase of angiotensin II [53]. Increasing angiotensin II could decreasingly change salivary grand functions. Intravenous infusion of angiotensin II (3–20 μg/h for 15 min) dose-dependently decreased saliva secretion of sheep parotid glands [54] and angiotensin II infusion (10–60 pmol/min) caused a blood flow reduction of rat submandibular glands [55] that was associated with a decrease of saliva secretion [56]. There is also an association between increased angiotensin II and inflammatory cytokine activation. Angiotensin II has the proinflammatory property to increase interleukin (IL)-6, which was confirmed after infusion of angiotensin II in humans [57].

#### 3.2.3. Salivary Gland Inflammation

Sialadenitis associated with acute or chronic salivary gland dysfunction is one of causes of xerostomia [58]. Patients with sialadenitis usually have the sensation of xerostomia due to reduced saliva secretion [59]. Chern et al. [60] reported a case of parotitis and submandibular gland sialadenitis in COVID-19 patients as well as a case of isolated parotitis. As Huang et al. [43] characterized the heterogeneous inflammatory responses of human salivary glands to SARS-CoV-2 infection, parotitis is one of clinical presentations of COVID-19 patients [61]. Wang et al. [62] hypothesized that SARS-CoV-2 infection could cause acute and chronic sialadenitis as follows. SARS-CoV-2 binds to ACE2 present in the duct epithelial cells of salivary glands, fuses with them, replicates and lyses the cells, producing inflammatory lesions in salivary glands. If the inflammation occurs in major salivary glands, their secretory functions would be disturbed, resulting in xerostomia. Although the acute inflammatory damages of salivary glands are repaired by fibroblast proliferation and fibrous connective tissue formation, the fibrosis repairments would cause chronic sialadenitis, inducing persistence of xerostomia.

A significant number of COVID-19 survivors (prevalence of 37.7%) developed salivary gland ectasia [16] that was considered to reflect the hyperinflammatory response to SARS-CoV-2 infection because of significant relations with C-reactive protein and lactate dehydrogenase levels at hospital admission. The main ductal ectasia of salivary gland is one of histopathological changes in Sjögren’s syndrome and infectious diseases [63].

#### 3.2.4. Zinc Deficiency

Zinc concentrations in serum are significantly lower in COVID-19 patients at hospitalization than healthy controls and the zinc-deficient patients show prolonged hospital stay and increased mortality [64]. Serum zinc deficiency defined as <70 μg/dL was detected in 79.6% of COVID-19 patients who were admitted to ICU and required invasive mechanical ventilation [65]. Yasui et al. [66] determined serum zinc levels of COVID-19 patients on the first day of hospitalization and 2–3 days later. Consequently, zinc deficiency was observed in 85.7% of patients with severe disease and in 13.6% of patients with mild to moderate disease. They also followed up with COVID-19 patients who were administered to ICU, treated with enteral nutrition delivered from the tube inserted through the nose and finally discharged from hospital. Serum zinc concentrations of the patients were found to maintain below or near the cut-off concentration of zinc deficiency for 4 weeks after disease onset. Other studies similarly indicated that serum zinc levels were reduced in COVID-19 patients [67,68] and that such a reduction was associated with an increase of COVID-19 severity [69].

Tanaka [70] compared salivary gland functions and zinc levels of 93 non-COVID-19 patients with xerostomia and/or hypogeusia, and consequently revealed that reduced saliva secretion correlated to zinc deficiency. Salivary flow rates of patients with confirmed or suspected zinc deficiency were less than those of controls in both parotid and submandibular glands (especially more significant in submandibular glands). Ishii et al. [71] prepared rat models of chronic zinc deficiency by feeding zinc-deficient diets and acute zinc deficiency by administrating zinc chelator dithizone. They found that the granule production in granular duct cells was decreased in rats with chronic zinc deficiency and that the degranulation of the granular duct cells and acinar cells in response to acetylcholine hydrochloride was strongly inhibited in rats with acute and chronic zinc deficiency. Their electron microscopic analysis of submandibular glands indicated that zinc was localized at the membrane surface, granules and vesicles of glandular epithelial cells and in the pits of myoepithelial cells. When rats were fed zinc-deficient and low-zinc diets for 6 weeks, the resultant zinc deficiency significantly reduced the activity of carbonic anhydrase in submandibular glands [72], suggesting that this enzyme is responsible for saliva secretion. Carbonic anhydrase is a zinc-metalloenzyme with the activity depending on zinc. If in vivo zinc levels decrease, the zinc concentrations in salivary glands should be lowered. A decrease of zinc content reduces the activity of carbonic anhydrase localized in acinar cells of submandibular glands [73] and parotid glands [74], thereby affecting their saliva secretion functions. When the zinc-deficient state continues, xerostomia would persist for weeks or months after recovery from COVID-19.

#### 3.2.5. Cranial Neuropathy

The autonomic nervous system innervates salivary glands to regulate their secretary functions by parasympathetic and sympathetic nerves being in contact with acinar, ductal and myoepithelial cells [46]. Parasympathetic stimulation induces secretion of a large volume of serous saliva from parotid glands and partly from submandibular glands, while sympathetic stimulation promotes production of protein-rich mucous saliva by sublingual and submandibular glands. Milovanovic et al. [75] assessed autonomic nervous system functions in the early phase of COVID-19 and demonstrated that parasympathetic, sympathetic or both nervous systems were functionally disordered in 51.5% and 76.7% of patients with severe and mild COVID-19, respectively. SARS-CoV-2, with the neurotropic and neuro-invasive properties, could interfere with parasympathetic and sympathetic innervation of salivary glands by the direct invasion of neural parenchyma or via the retrograde axonal transport.

In salivary reflexes, taste, mechanical smell and pungent stimuli generate afferent signals in fibers of the facial nerve (cranial nerve VII, CN VII), the glossopharyngeal nerve (cranial nerve IX, CN IX) and the trigeminal nerve (cranial nerve V, CN V) [46]. The nucleus of the solitary tract is innervated by CN VII and CN IX, and sends interneurons to the salivary centers, superior and inferior salivary nuclei. Efferent nerve fibers from the salivary nuclei conduct efferent signals via the chorda lingual nerve to the submandibular ganglion, and then postganglionic nerves innervate submandibular and sublingual glands, while parotid glands are supplied by efferent fibers in the glossopharyngeal (tympanic branch) nerve via the otic ganglion. Neuropathy of cranial nerves, CN VII, CN IX and CN X, is involved in COVID-19 [76]. Among them, CN VII neuropathy especially leads to the clinical presentation of xerostomia and taste dysfunction [77].

#### 3.2.6. Intercurrent Taste Dysfunction

A number of COVID-19 patients and survivors complain of xerostomia together with taste dysfunction as described in the Section 3.1.5. The co-occurrence and co-persistence of these two oral symptoms suggest that xerostomia may occur secondary to taste dysfunction and vice versa [4,78].

Taste substances dissolved in saliva interact with taste receptors of taste buds to not only mediate taste perception by triggering the first step of taste signal transduction but also stimulate secretion of major salivary glands via the gustatory-salivary reflex. Salivary reflexes are afferently innervated by CN VII, CN IX and CN V [79]. Neuro-invasive SARS-CoV-2 causes neuropathy of these cranial nerves involved in the taste perception system and the gustatory–salivary reflex, inducing taste dysfunction and xerostomia. Salivary flow and composition are possibly altered by SARS-CoV-2 infection of salivary glands. Such alterations lead to both xerostomia and taste dysfunction [80].

Secretion from submandibular and sublingual glands is also promoted by the smell of food. Olfactory dysfunction that frequently occurs in COVID-19 patients may additionally contribute to xerostomia with the subsequent taste dysfunction.

#### 3.2.7. Comorbidities and Medications

As characterized in the Section 3.1.6., the most common comorbidities of COVID-19 patients and survivors are diabetes mellitus and hypertension, followed by chronic pulmonary disease, thyroid disease and chronic kidney disease. Htun et al. [81] reported that among COVID-19 patients with comorbidities, hypertension, diabetes mellitus and heart diseases showed prevalences of 58.3%, 29.8% and 26.2%, respectively. Xerostomia has been referred to as a frequent complaint of diabetic patients [82]. COVID-19 xerostomia may be partly due to comorbidities represented by diabetes mellitus.

Xerostomia occurs as a side effect of medications, which are categorized into the drugs used for treating COVID-19 and the drugs prescribed for comorbidities. In the former category, chloroquine/hydroxychloroquine, combined lopinavir and ritonavir, and interferon-β potentially induce dry mouth although all of them exert anti-viral effects. In the latter category, many prescribed drugs cause oral side effects, most frequently xerostomia or dry mouth, followed by taste dysfunction [83]. Xerostomia-inducing drugs include antihypertensive (β-blockers, α-agonists, calcium channel blockers and ACE inhibitors), sedative/anxiolytic (benzodiazepines), antidepressant, diuretic, antihistamine and anticholinergic or parasympatholytic drugs [84]. Since hypertension is one of the most common comorbidities in COVID-19 patients and survivors, it is highly probable that they are using antihypertensive drugs, which may be an iatrogenic cause for xerostomia.

### 3.3. Possible Therapy

Since no methods have yet to be universally accepted for the treatment of COVID-19 xerostomia, the therapy for xerostomia and hyposalivation in non-COVID-19 cases may be applicable to COVID-19 patients and survivors.

As a symptomatic therapy, saliva substitutes are usable for relieving the subjective feeling of mouth dryness and lubricating the oral cavity. Promising sialagogues are parasympathomimetic drugs (nonselective muscarinic agonist pilocarpine and muscarinic-3 receptor selective agonist cevimeline), taste response-stimulators (ascorbic and malic acid) and mechanical stimulators of parotid glands (sugar-free chewing gum). When cevimeline and pilocarpine were orally administered to patients with xerostomia in post-radiotherapy, Sjögren’s syndrome and sialosis/drug-induced groups, both drugs increased salivary flow rates and decreased the symptoms relating to xerostomia [85,86]. However, such parasympathomimetic agents, especially pilocarpine, have side effects of excess sweating and urinary frequency.

Since zinc is involved in the secretory functions and the growth of myoepithelial and glandular epithelial cells of salivary glands, acute and chronic zinc deficiency has been shown to significantly reduce saliva secretion of rats [71]. While zinc-metalloenzyme carbonic anhydrase is responsible for saliva production and secretion, the expression of its isozyme II was found to decrease in submandibular glands of zinc-deficient rats with reduced saliva secretion [87]. If zinc deficiency is responsible at least in part for the pathogenesis of COVID-19 xerostomia, zinc supplementation is expected to improve the symptom. Administration of zinc acetate (15 mg zinc/day orally for 5 weeks) increased the flow rates of stimulated saliva from parotid glands of healthy adult subjects [88]. When zinc sulfate (300 mg/day orally for 6 months) was prescribed for patients with oral symptoms, xerostomia and taste dysfunction was relieved or improved in 57.9% and 72.7% of cases, respectively [70]. Kim et al. [89] demonstrated that mouth rinsing with 0.25% ZnCl_2_ solution for 3 min (not affecting taste) increased both unstimulated and mastication-mediated stimulated saliva secretion in healthy subjects and hyposalivation patients. Since metabotropic zinc receptor ZnR/GPR39 is expressed in human submandibular gland cells, zinc may induce saliva secretion via ZnR/GPR39 independently of muscarinic receptor signaling. Cellular intake of zinc is possibly enhanced by using ionophores. The supplementation combined with zinc ionophores to enhance the cellular intake of zinc, such as chloroquine, hydroxychloroquine and bioflavonoids (quercetin or (–)-epigallocatechin-3-gallate), would be more effective for COVID-19 xerostomia.

## 4. Conclusions

Similar to taste dysfunction, xerostomia is one of the most prevalent and persistent oral symptoms associated with COVID-19. Despite a close association with COVID-19, xerostomia, dry mouth and hyposalivation tend to be overlooked in COVID-19 patients and survivors, unlike ageusia, dysgeusia and hypogeusia. The symptom characterization of xerostomia is not necessarily consistent with that of taste dysfunction, suggesting a pathogenesis specific to COVID-19 xerostomia such as salivary glands expressing the SARS-CoV-2 cellular entry-relevant proteins. Although mouth dryness per se is not life-threating, it has a negative impact on the oral health-related quality of life of COVID-19 survivors because saliva plays important roles in taste, mastication, swallowing, food digestion and speech. Hyposalivation leads to taste dysfunction, mastication difficulty, dysphagia, malnutrition and speech problems. Xerostomia is also a potential risk factor to increase the occurrence of dental caries, periodontal disease, oral mucosal ulceration, halitosis and oral candidiasis. More attention should be paid to xerostomia, dry mouth and hyposalivation of patients infected with SARS-CoV-2. In addition, development of an effective therapy for such symptoms is an urgent issue, which should be tailored individually because the pathogenesis of COVID-19 xerostomia is likely to be multifactorial depending on patients.

## Figures and Tables

**Table 1:** Xerostomia, dry mouth and hyposalivation associated with COVID-19.

Patients and Diagnosis	Disease Severity	Country or Ethnicity	Number of Patients	Age (Year), Mean or Median (Range)	Female (%)	Oral Symptoms	Prevalence (%)	Comorbidities	Reference
Patients diagnosed by RT-PCR test	NR	Italy (European 88%, Asian 12%)	50	37.7 (18–65)	40.0	Xerostomia Taste dysfunction in the early phase Xerostomia Taste dysfunction after 15 days from RT-PCR test negativity	32.0 70.0 2.0 8.0	DM (6.0%) HT (16.0%) CRD (10.0%) TD (8.0%) Asthma (14.0%)	Freni et al. [6]
Non-hospitalized patients diagnosed by RT-PCR test	Mild	Israel	128	36.3 (18–73)	54.7	Dry mouth Taste dysfunction in the early phase Changes in Sweet taste Salty taste Sour taste	56.3 52.3 47.7 42.2 41.4	DM (2.3%) HT (6.3%) TD (3.1%) Asthma (1.6%)	Biadsee et al. [7]
Patients diagnosed by real-time RT- PCR test	Mostly mild to moderate	Italy	111	57 (48–67)	47.7	Xerostomia Dysgeusia in the early phase	45.9 59.5	DM (9.0%) HT (26.1%) CPD (9.9%)	Fantozzi et al. [14]
Hospitalized patients	NR	Italy	20	69.2	45.0	Xerostomia Taste impairment Swallowing difficulty Burning sensation during hospitalization	30.0 25.0 20.0 15.0	DM (15%) HT (40%) TD (25%)	Sinjari et al. [15]
Hospitalized patients diagnosed according to the official guideline and by SARS-CoV-2 nucleic acid detection	NR	China	108	52.0 (one female, age data missed)	51.9	Dry mouth Amblygeustia in the early phase	46.3 47.2	NR	Chen et al. [8]
Hospitalized patients diagnosed by real-time RT- PCR test	Moderate to severe	Italy	122	62.5	24.6	Dry mouth Salivary gland ectasia Taste alteration after 95–132 days from hospital discharge	24.6 37.7 42	DM (26.7%) HT (40.0%) CPD (20.0%) CKD (6.7%)	Gherlone et al. [16]
Non-hospitalized patients diagnosed by PCR test	Mild to moderate	Egypt	573	36.2 (19–50)	71.2	Xerostomia Halitosis Ulcerations in patients recovered from COVID-19	47.6 10.5 20.4	Medically free and not suffering from any oral manifestations before disease onset	AbuBakr et al. [17]
Patients diagnosed by PCR test who completed treatment at hospital or home at least 2 weeks ago	Mild to moderate	Turkey	107	34.5 (13–70)	47.7	Xerostomia Hyposalivation Taste impairment after 2–4 weeks from hospital discharge	40.2 18.5 56.1	Neither comorbidities nor medications to affect saliva flow/amount	Omezli & Torul [18]
Hospitalized patients diagnosed by RT-PCR test and lung CT scan	NR	Iran	10	42.6 (19–49)	50	Xerostomia 3–4 days before prodromal symptoms such as fever and respiratory complications	60	GD (30%)	Fathi et al. [19]
Non-hospitalized patients diagnosed by RT-PCR test	Mild	Israel	97	37.5 (19–74)	55.7	Xerostomia Taste dysfunction at the first survey Xerostomia Taste dysfunction after 191–253 days from RT-PCR test negativity	61.9 67.0 14.4 25.8	NR	Biadsee et al. [20]
Hospitalized patients diagnosed by RT-PCR test	NR	Egypt	58	18–46	46.6	Dry mouth Taste dysfunction during disease	39.7 34.5	NR	El Kady et al. [21]
Patients diagnosed by PCR test	Ambulatory (35.0%), severe (41.0%), Critical (24.0%)	Colombia	100	49 (37.8–55.3)	53.0	Xerostomia Ageusia after 219 days from symptom onset	26.0 53.0	DM (15.0%) HT (17.0%) CPD (1.0%) TD (12.0%) CKD (3.0%)	Anaya et al. [22]

NR: Not reported. RT-PCR: Reverse transcription polymerase chain reaction. DM: Diabetes mellitus. HT: Hypertension. CRD: Chronic respiratory disease. CPD: Chronic pulmonary disease. TD: Thyroid disease. CKD: Chronic kidney disease. GD: gastrointestinal disorder. Demographic data and disease severity at baseline.

## Data Availability

Not applicable.
